# *Streptococcus iniae* Discitis in Singapore

**DOI:** 10.3201/eid1009.040029

**Published:** 2004-09

**Authors:** Tse Hsien Koh, Asok Kurup, John Chen

**Affiliations:** * Singapore General Hospital, Singapore

**Keywords:** letter, β-hemolytic streptococcus

**To the Editor:** *Streptococcus iniae* is a well-recognized fish pathogen that can cause meningoencephalitis in tilapia and trout (*1*) and necrotizing myositis in red drum (*2*). We describe the first known human case of *S. iniae* infection in Singapore. This is the second report of spinal infection with this bacterium; however, commercial kits may misidentify *S. iniae*.

The first cases of *S. iniae* infection in humans were reported in Toronto, Canada, in 1995–1996 and included eight patients with bacteremic hand cellulitis and one patient with endocarditis, meningitis, and arthritis (*3*). Two additional cases were discovered retrospectively, a patient in Ottawa, Canada, with septic arthritis of the knee and a patient from Texas with bacteremic cellulitis. At least two more strains have been isolated from patients in Vancouver, Canada (*4*). Recently, Lau et al. described two cases of infection in Hong Kong. The first patient had bacteremic cellulitis; the second is recognized as the first patient with *S. iniae* osteomyelitis of the spine (*5*).

A 73-year-old female Chinese healthcare worker was admitted on October 5, 2003, to Singapore General Hospital. Her symptoms were fever for 3 days before admission and lower back pain that had progressively worsened for the past 2 months, causing her to become bedridden. She was ambulatory before the back pain started and had no history of a fall or injury to the back.

Upon examination, the patient's temperature was 37.1°C. She did not appear septicemic and was hemodynamically stable. No evidence of cellulitis was found, and neurologic examination of the upper limbs showed no abnormalities. Movement and strength of both lower limbs were limited by pain. Reflexes and plantar responses were normal, and no focal tenderness over the spine was found; chest x-ray results were normal. Laboratory tests showed the following: leukocytes 12.91 x 10^9^/L, hemoglobin 9.9 g/dL, platelets 261 x 10^9^/L, serum albumin 20 g/L, bilirubin 17 µmol/L, alkaline phosphatase 132 U/L, alanine transaminase 16 U/L, and aspartate transaminase 23 U/L. Renal function tests were within normal limits. The erythrocyte sedimentation rate was 115 mm/h, and C-reactive protein was 88.4 mg/L. No bacteria were grown from blood cultures. Treatment with empiric intravenous cefazolin was started.

Magnetic resonance imaging (Figure) of the patient's lumbar spine revealed discitis and osteomyelitis at the L3/L4 level, with associated epidural and paraverterbral abscesses. Anterior drainage of the abscesses and fusion with an iliac crest graft were performed on day 5 of hospitalization. Tissue samples obtained during this operation showed pus cells and gram-positive cocci. Cultures grew a &beta;-hemolytic streptococcus which did not group with Lancefield groups A, B, C, D, F, or G antisera and was positive for pyrrolidonylarylamidase, negative for bile-esculin, and failed to grow in 6.5% NaCl. The API 20 Strep (bioMérieux, Marcy l'Etoile, France) identified the isolate as *S. dysgalactiae* subsp. *equisimilis* (profile number 4563117). Because *S. dysgalactiae* subsp. *equisimilis* are negative for pyrrolidonylarylamidase, we sequenced the 16S rRNA gene, which was identical to that of *S. iniae* ATCC29178 isolated in 1976 from a subcutaneous abscess of a captive Amazonian dolphin (*6*). The isolate was susceptible to penicillin, chloramphenicol, clindamycin, and vancomycin by antimicrobial disk diffusion tests (*7*).

**Figure:**
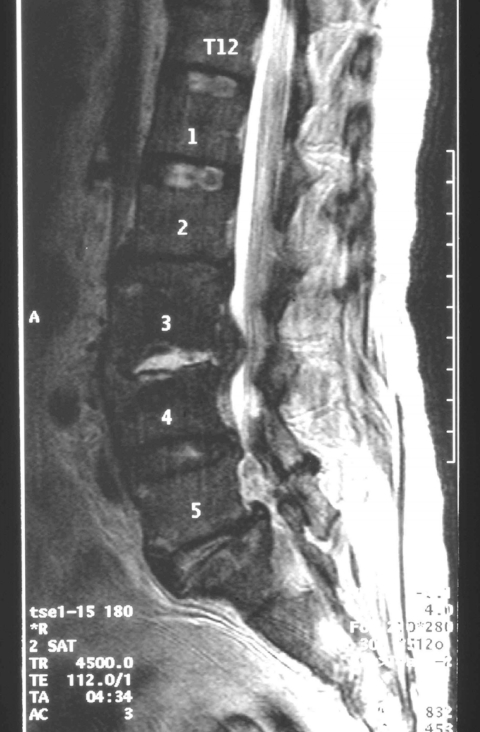
Magnetic resonance imaging scan of spine showing features compatible with discitis involving L3/L4 with surrounding paravertebral and psoas inflammation and epidural abscess.

The patient was asked specifically about exposure to fresh fish and other aquatic creatures before she became ill. She regularly prepared fresh fish bought from the local market and had sustained superficial cuts on her fingers before her back pain started. Though she did not recall any upper limb infection or previous septicemic episode, she likely had a transient bacteremia which spread to her lumbar spine.

The patient was given 2.4 g of intravenous penicillin every 4 hours for 6 weeks; she showed clinical improvement with decreasing erythrocyte sedimentation rate and C-reactive protein levels. At the time of discharge she was ambulatory with a walker.

Most confirmed cases of *S. iniae* infection to date have occurred in persons of Asian, predominantly Chinese, ethnicity. This phenomena is thought to result from a cultural preference for fresh, whole fish in cooking. Although *S. iniae* infection in fish is often associated with aquaculture, human infection has not been noted in fish-farm workers. A decline in immune function may be important in pathogenesis since most cases reported have been in elderly persons (mean age 70 years, range 40–81 years) (*5*).

The diagnosis of *S. iniae* infection may be missed because commercial identification kits do not include *S. iniae* in their databases. The Hong Kong and Singapore isolates were identified by the API 20 Strep system as *S. dysgalactiae* subs. *equisimilis*, and some Canadian isolates were identified by the VITEK system (bioMérieux, Marcy l'Etoile, France) as *S. uberis* (*3*). This problem may be compounded because physicians who are not familiar with the infection would not question the patient about exposure to fresh fish.

Apart from *S. iniae*, few other &beta;-hemolytic streptococci are pyrrolidonylarylamidase positive. &beta;-hemolytic enterococci and *S. pyogenes* are groupable with antisera to Lancefield group D and A antigens, respectively, and are easily identified by routine laboratory methods (*8*). *S. porcinus* is a rare human pathogen resistant to bacitracin, which produces a positive CAMP reaction and may react with antisera to Lancefield group B antigen (*9*).

All &beta;-hemolytic streptococci that are ungroupable by Lancefield antisera should be tested for pyrrolidonylarylamidase. If this test is positive, the patient should be questioned about exposure to fresh fish, and identification of the isolate should be confirmed by molecular means.

A retrospective review of laboratory records showed that we had isolated &beta;-hemolytic streptococci nongroupable with Lancefield groups A, B, C, D, F, or G antisera only five times in the last 2 years. Most of these were from respiratory sources and unlikely to be *S. iniae*. Therefore, we believe that *S. iniae* infection in Singapore is uncommon. However, the geographic range of this emerging zoonosis is likely to increase as clinicians and microbiologists become more aware of this pathogen.

The 16S rRNA gene sequence for this isolate has been submitted to GenBank (accession number AY581891).
